# COVID-19 with neck cellulitis: an under recognized acute presentation

**DOI:** 10.1590/0037-8682-0091-2024

**Published:** 2024-05-20

**Authors:** Sravani Mannuru, Márcio Luís Duarte, Leonardo Furtado Freitas

**Affiliations:** 1 Neuroradiology Division, Radiology Department, University of Iowa Hospitals and Clinics, Iowa City, IA, USA; 2 Universidade de Ribeirão Preto - Campus Guarujá, Guarujá, SP, Brasil.

**FIGURE 1:**
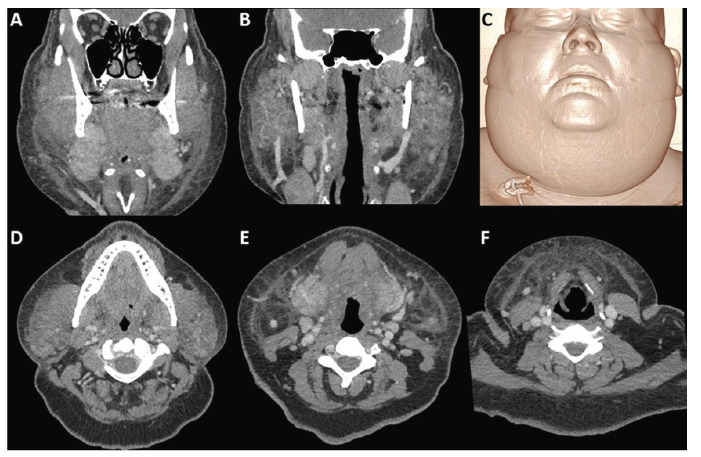
Coronal **(A-B)** and axial **(D-F)** CT neck with contrast. 3D volume rendering reformatting **(C)**. Diffuse fatty stranding of the lateral and anterior neck, with thickening of the platysma muscles. The process extended to the parapharyngeal, parotid, and submandibular spaces, with reactive sialadenitis. Multiple sub-centimeter lymph nodes and mucosal/submucosal edema of the oropharynx were observed. No drainable/organized collections identified.

A 51-year-old male with a recent COVID-19 diagnosis was presented to the emergency room with sudden neck swelling. Computed tomography (CT) of the neck revealed diffuse cellulitis with reactive sialadenitis and lymphadenopathy (Figure 1). In this case, there were no drainable collections or skin rashes. Otolaryngology was consulted, and the patient was started on nirmatelvir/ritonavir, amoxicillin/clavulanate, sialagogues, and a heating pad.

This unusual acute presentation arises from complications directly or indirectly associated with COVID-19, leading to systemic inflammatory response syndrome^1^. Some instances of deep neck abscesses linked to COVID-19 have been reported^2^, which can progress rapidly and pose life-threatening risks, underscoring the critical importance of early diagnosis^3^. Various factors, such as dental infections, trauma, tonsillitis, foreign bodies, and head and neck malignancies can precipitate deep neck infections and abscesses^3^. Immunocompromised patients with underlying systemic conditions, such as diabetes mellitus and chronic renal failure are at a higher risk of complications^2^. Although there are limited case reports linking COVID-19 with sudden neck swelling, deep neck abscesses can persist even after COVID-19 treatment completion^2^. Notably, SARS-CoV-2 infection can predispose individuals to secondary bacterial superinfections^4^. A meta-analysis conducted by Langford et al. involving 3,338 patients identified secondary bacterial infections in 6.9% of COVID-19 cases^5^. Identifying this unusual acute clinical presentation of COVID-19 may facilitate early detection and improve treatment efficacy.
